# Evaluating the efficacy and safety of brolucizumab versus aflibercept on visual and anatomical outcomes in diabetic macular edema: a retrospective comparative study

**DOI:** 10.1007/s00417-026-07211-6

**Published:** 2026-04-17

**Authors:** Ehab Mohamed Elsayed Saad, Wafaa Kamel Abdelmoneim Abdelmaged, Ahmed Alyan, Sherif A. Dabour, Ahmed Abdelshafy Tabl

**Affiliations:** 1 Ophthalmology Department, Faculty of Medicine, Benha University, Benha, Egypt; 2 Internal Medicine Department, Faculty of Medicine, Benha University, Benha, Egypt; 3 Ophthalmology Department, Faculty of Medicine, Zagazig University, Zagazig, Egypt

**Keywords:** Diabetic macular edema, Brolucizumab, Aflibercept, Anti-VEGF therapy

## Abstract

**Purpose:**

The efficacy and safety of Aflibercept (AFL) 2 mg versus Brolucizumab (BRZ) 6 mg in diabetic macular edema (DME) were evaluated in this retrospective comparative research.

**Patients and methods:**

Thirty-eight eyes with DME for > 18 months were retrospectively reviewed and divided equally. Group A received three loading doses of intravitreal BRZ (6 mg) and Group B received five loading doses of intravitreal AFL (2 mg), followed by pro re nata (PRN) dosing per OCT-detected persistent macular edema. The central macular thickness (CMT) change from baseline was the main outcome measure. Injection count, complication rate, and best-corrected visual acuity (BCVA) were the secondary objectives.

**Results:**

Thirty-eight eyes were analyzed (19 per group). At six months, BCVA and CMT improvements were comparable (*p* > 0.05). At 12 and 18 months, BRZ achieved significantly greater BCVA gain (0.17 ± 0.08 vs. 0.34 ± 0.11 logMAR, *p* < 0.001) and greater CMT reduction (239.5 ± 12.5 μm vs. 263.2 ± 19.8 μm, *p* < 0.001) compared with AFL. The BRZ group had a considerably lower mean total number of injections (5 ± 1.2 vs. 9 ± 1.3, *p* < 0.001), representing a 44% reduction in injection burden. A mild, non-vision-threatening retinal vasculitis occurred in one BRZ patient (5.3%, *p* = 0.4866).

**Conclusion:**

BRZ provided superior visual and anatomical outcomes compared with AFL at 12 and 18 months, with significantly fewer injections. Both agents demonstrated acceptable safety profiles. With a lower treatment burden and long-lasting effectiveness in practical situations, our results validate BRZ as a viable and long-lasting therapy option for DME.

## Key messages

*****What is known***:**

- Diabetic macular edema (DME) is a leading cause of vision loss in working-age adults, and anti-VEGF therapy is the standard of care.
- Brolucizumab (BRZ) and aflibercept (AFL) have demonstrated efficacy in clinical trials, but standard clinical practice comparative data on injection burden, long-term visual and anatomical outcomes, and safety are limited.

*****What is new***:**

- This study provides standard clinical practice comparative data on BRZ and AFL, including injection burden, long-term visual and anatomical outcomes, and safety.
- BRZ achieved superior visual (BCVA) and anatomical (CMT) improvements at 12 and 18 months compared with AFL.
- BRZ required 44% fewer intravitreal injections over 18 months, with a low incidence of manageable ocular inflammation.

## Introduction

Diabetic macular edema (DME) is the main cause of visual impairment in working-age persons with diabetes. It results from persistently elevated blood sugar, which causes oxidative stress and an increase in vascular endothelial growth factor (VEGF), which in turn causes more retinal vascular leakage, fluid accumulation, and macula thickening [1, 2]. Globally, diabetes affects approximately 536.6 million adults, with 5.5% developing OCT-confirmed DME, a burden projected to grow [3].

International guidelines recommend anti-VEGF treatments, such as faricimab, ranibizumab, aflibercept (AFL), and brolucizumab (BRZ), as the standard therapy for center-involving DME [3]. Phase III trials (KESTREL and KITE) showed BRZ (6 mg) achieves non-inferior visual acuity gains and greater reductions in central subfield thickness as opposed to AFL (2 mg), with extended dosing intervals up to 12–16 weeks, though with slightly higher intraocular inflammation rates [4, 5]. Real-world investigations confirm BRZ’s efficacy, with treatment-naïve DME eyes gaining + 6.4 ETDRS letters and longer injection intervals (11.3 vs. 6.5 weeks for AFL) [6]. Some eyes achieved 16-week treatment-free intervals after one BRZ injection [7] and a 2024 meta-analysis supported its reduced injection frequency [8]. However, real-world data report intraocular inflammation in 2.4–16% of cases, with rare vasculitis manageable by early intervention [9, 10]. A cohort study noted anatomical improvements and + 2 ETDRS letters one month post-BRZ injection [11].

Despite these realizations, there is little empirical data about the relative safety and effectiveness of BRZ and AFL in DME patients who have early persistent fluid, a subset that may react differently to therapy. By assessing the visual and anatomical results, injection load, and safety profiles of various medicines, this retrospective study sought to close this gap and provide more individualized and efficient DME care in clinical practice.

## Materials and methods

### Study design and participants

38 eyes from 38 patients treated at Benha University Hospital between January 2023 and July 2024 for center-involving diabetic macular edema (DME) were included in this retrospective, comparative research. Patients were divided into two groups according to the kind of intravitreal anti-VEGF therapy they received: Group A (BRZ) consisted of 19 eyes treated with intravitreal brolucizumab (6 mg/0.05 mL), and Group B (AFL) consisted of 19 eyes treated with intravitreal aflibercept (2 mg/0.05 mL).

The study was registered at ClinicalTrials.gov in (2024-01-01) under the identifier NCT07096713.

The study protocol was reviewed and approved by the Ethics Committee of the Faculty of Medicine, Benha University (Approval Code: Rc 5_1_2025).

### Inclusion criteria

Eyes with center-involving DME verified by spectral-domain optical coherence tomography (SD-OCT) with a central macular thickness (CMT) more than 350 μm were considered eligible. All included eyes had stable glycemic control and were followed for at least 18 months after initiation of anti-VEGF therapy. Patients were required to have adequate media clarity to allow high-quality fundus and OCT imaging and reliable visual acuity assessment.

### Exclusion criteria

Excluded were eyes having severe media opacities that affected retinal imaging, such as thick cataracts, corneal haze, or vitreous hemorrhage. Additional exclusion criteria included the presence of tractional maculopathy or vitreomacular interface abnormalities on OCT, history of intraocular inflammation, proliferative diabetic retinopathy requiring panretinal photocoagulation within the past six months, and systemic inflammatory or autoimmune disorders (e.g., systemic lupus erythematosus). Eyes that had undergone intraocular surgery or received intravitreal injections for other indications within the previous six months were also excluded.

### OCT imaging and visual acuity assessment

The Spectralis SD-OCT system (Heidelberg Engineering, Germany) was used for retinal imaging. The macular cube protocol (20° × 20°; 49 B-scans) centered on the fovea was employed for quantitative assessment of central macular thickness.

At each visit, the Early Treatment Diabetic Retinopathy Study (ETDRS) charts were used to assess best-corrected visual acuity (BCVA) at a distance of 4 m. The BCVA was then converted to logarithm of the minimum angle of resolution (logMAR) values for analysis.

### Treatment protocol

As this was a retrospective, comparative analysis, treatment regimens reflected the standard clinical practice during the study period rather than a controlled trial protocol.

Following an initial set of three loading injections, patients in Group A received intravitreal BRZ (6 mg/0.05 mL) every eight weeks. On the other hand, in accordance with the authorized labeling and hospital procedure for DME, patients in Group B received intravitreal AFL (2 mg/0.05 mL) every four weeks for five loading doses. Based on OCT evidence of chronic or recurrent macular edema and/or loss of ≥ 5 ETDRS letters, both groups were then kept on a pro re nata (PRN) regimen.

Because of these inherent differences in approved dosing intervals, the regimens were not equivalent by design; however, this allowed a clinically relevant comparison of efficacy and injection burden across commonly used anti-VEGF agents in routine practice.

### Data collection

Retrospective data extraction was done from baseline and 6-, 12-, and 18-month visits in medical records. BCVA, intraocular pressure (IOP), fundus and slit-lamp examination results, CMT readings, and the incidence of ocular or systemic adverse events were among the variables that were recorded. Systemic data such as glycated hemoglobin (HbA1c) and blood pressure were also collected to assess their potential correlations with anatomical and functional outcomes.

### Study outcomes

The central macular thickness (CMT) change from baseline to month 18 was the main result.

Changes in BCVA (ETDRS letters), the total amount of intravitreal injections, and the frequency of systemic or ocular problems including thromboembolic events, retinal vasculitis, or uveitis were examples of secondary outcomes.

### Statistical analysis

Version 2.6.26 of the Jamovi software was used to analyze the data. The cohort’s clinical and demographic features were compiled using descriptive statistics. The Shapiro-Wilk test evaluates the data distribution and presents continuous variables as either mean ± SD or median (interquartile range [IQR]). Counts and percentages are used to express categorical variables.

The independent-samples t-test or Mann-Whitney U test for continuous variables and the Chi-square or Fisher’s exact test for categorical variables, respectively, were used to compare treatment groups. In particular, the Shapiro-Wilk test was used to assess the injection count distribution, and the independent-samples t-test for normally distributed data or the Mann-Whitney U test for non-normal distributions were used to compare groups.

Depending on the symmetry of the variable distribution, either Pearson’s or Spearman’s coefficients were used to analyze linear correlations. A multivariable linear regression model was built using patient age, sex, duration of diabetes, intraocular pressure (IOP), baseline BCVA, central macular thickness (CMT), and total number of injections as covariates in order to determine predictors of best-corrected visual acuity (BCVA) at six months. The model coefficients (β) were presented together with the corresponding p-values and 95% CI. A two-tailed *p* value < 0.05 was considered statistically significant.

### Sample size determination

A priori sample estimation was performed using G*Power version 3.1.9.7, guided by persistent macular fluid rates reported in the literature [3, 8]. Employing a two-sided chi-square test with a power of 80% and Type I error rate of 5%, the analysis indicated a minimum sample size of 34 eyes (17 per group) to detect significant intergroup differences. To accommodate the potential data attrition inherent in retrospective designs, the final target sample was inflated by 10%, yielding 38 eyes (19 per group). Post-hoc analysis confirmed that the study retained adequate statistical power (82%) to detect differences in persistent fluid presence between the cohorts.

### Ethical approval

The Ethics Committee of Benha University’s Faculty of Medicine examined and approved the study protocol (Approval Code: Rc 5_1_2025). In compliance with national and institutional ethical guidelines for retrospective research, the need for formal informed permission was not required. To protect participant confidentiality, all data were completely anonymised before analysis. NCT07096713 was the study’s registration number on ClinicalTrials.gov.

## Results

### Baseline characteristics

Group A (BRZ, *n* = 19) and Group B (AFL, *n* = 19) were the two equal groups of 38 eyes from 38 patients with center-involving DME. Tables 1 and 2 provide a summary of the demographic and baseline clinical features.

**Table 1:** Baseline Demographic and Clinical Characteristics

	Group A (*N* = 19)	Group B (*N* = 19)	Total (*N* = 38)	*P*
Age				0.0771
Mean ± SD	59.5 ± 5.4	54.3 ± 11.2	56.9 ± 9.1	
Range	49.0–69.0	22.0–70.0	22.0–70.0	
Sex				0.0232
Female	6.0 (31.6%)	13.0 (68.4%)	19.0 (50.0%)	
Male	13.0 (68.4%)	6.0 (31.6%)	19.0 (50.0%)	
Duration of diabetes mellitus				0.8641
Mean ± SD	12.4 ± 2.5	12.5 ± 3.1	12.4 ± 2.8	
Range	8.0–18.0	7.0–21.0	7.0–21.0	

SD: Standard Deviation

**Table 2:** Baseline Ocular Characteristics

	Group A (*N* = 19)	Group B (*N* = 19)	Total (*N* = 38)	*P*
Intraocular Pressure				0.4121
Mean ± SD	14.2 ± 3.0	13.5 ± 2.4	13.8 ± 2.7	
Range	10.0–22.0	10.0–18.0	10.0–22.0	
Best-Corrected Visual Acuity				0.9571
Mean ± SD	0.9 ± 0.6	1.0 ± 0.6	1.0 ± 0.6	
Range	0.2–1.8	0.1–1.8	0.1–1.8	
CMT-OCT µm				0.2661
Mean ± SD	359.3 ± 85.0	380.5 ± 85.2	369.9 ± 85.4	
Range	233.0–477.0	233.0–499.0	233.0–499.0	

CMT: Central Macular Thickness, OCT: Optical Coherence Tomography, SD: Standard Deviation

Group A and Group B had similar age distributions, with mean ages of 59.5 ± 5.4 and 54.3 ± 11.2 years, respectively (*p* = 0.077). Although a statistically significant sex difference was observed (*p* = 0.023), the two groups were similar with respect to diabetes duration (12.4 ± 2.5 vs. 12.5 ± 3.1 years, *p* = 0.864).

The groups’ baseline ocular characteristics were similar as well. Group A’s mean IOP was 14.2 ± 3.0 mmHg, whereas Group B’s was 13.5 ± 2.4 mmHg (*p* = 0.412). Baseline BCVA was comparable (*p* = 0.957). The inclusion of eyes with center-involving edema > 350 μm and similar anatomical severity at baseline was confirmed by the mean baseline CMT values of 359.3 ± 85.0 μm and 380.5 ± 85.2 μm for Groups A and B, respectively (*p* = 0.266).

### Injection frequency

As this was a retrospective comparative analysis, the two agents followed their respective clinically used dosing protocols. Group A received three intravitreal BRZ injections (6 mg) administered every 8 weeks (weeks 0, 8, and 16) as a loading phase, whereas Group B received five intravitreal AFL injections (2 mg) administered every 4 weeks (weeks 0, 4, 8, 12, and 16).

During the PRN phase, Group A required a mean of 2 additional injections (total = 5 ± 1.2), while Group B received 4 additional injections (total = 9 ± 1.3). Although this difference reflects the respective approved dosing intervals and drug durability rather than protocol inequality, it resulted in a 44% lower cumulative injection burden with BRZ over the 18-month follow-up period.

### Visual and anatomical outcomes

#### At 6 months

As shown in Table 3, both treatment groups demonstrated comparable functional and anatomical improvements. The mean best-corrected visual acuity (BCVA) was 0.40 ± 0.20 logMAR in the BRZ group and 0.50 ± 0.30 logMAR in the AFL group (*p* = 0.123). Mean central macular thickness (CMT) values were 286.1 ± 19.5 μm and 300.7 ± 55.3 μm, respectively (*p* = 0.284).

**Table 3:** Visual Acuity and Central Macular Thickness at 6-Month Follow-Up

	Group A (*N* = 19)	Group B (*N* = 19)	Total (*N* = 38)	*P*
Best-Corrected Visual Acuity- 6 m				0.1231
Mean ± SD	0.4 ± 0.2	0.5 ± 0.3	0.5 ± 0.2	
Range	0.2–0.7	0.1–1.0	0.1–1.0	
CMT-OCT µm − 6 m				0.2841
Mean ± SD	286.1 ± 19.5	300.7 ± 55.3	293.4 ± 41.6	
Range	236.0–301.0	231.0–391.0	231.0–391.0	

CMT: Central Macular Thickness, OCT: Optical Coherence Tomography, SD: Standard Deviation

#### At 12 months

There were statistically significant intergroup differences that favored BRZ, as shown in Table 4. With a BCVA improvement of 0.20 ± 0.10 logMAR, Group A showed more visual improvement than Group B, which showed a BCVA improvement of 0.40 ± 0.10 logMAR (*p* < 0.001). As a result, the mean CMT dropped from 270.1 ± 21.1 μm in Group B to 247.6 ± 13.6 μm in Group A (*p* < 0.001).

**Table 4:** Visual Acuity and Central Macular Thickness at 12-Month Follow-Up

	Group A (*N* = 19)	Group B (*N* = 19)	Total (*N* = 38)	*P*
Best-Corrected Visual Acuity- 12 m				< 0.0011
Mean ± SD	0.2 ± 0.1	0.4 ± 0.1	0.3 ± 0.1	
Range	0.1–0.4	0.1–0.5	0.1–0.5	
CMT-OCT µm − 12 m				< 0.0011
Mean ± SD	247.6 ± 13.6	270.1 ± 21.1	258.8 ± 20.9	
Range	231.0–271.0	234.0–301.0	231.0–301.0	

CMT: Central Macular Thickness, OCT: Optical Coherence Tomography, SD: Standard Deviation

#### At 18 months

As presented in Table 5, these functional and anatomical advantages were sustained through the end of follow-up. Group A maintained superior visual outcomes (BCVA = 0.17 ± 0.08 logMAR) compared with Group B (0.34 ± 0.11 logMAR; *p* < 0.001). Similarly, CMT remained significantly lower in Group A (239.5 ± 12.5 μm) than in Group B (263.2 ± 19.8 μm; *p* < 0.001), underscoring the prolonged efficacy of BRZ therapy.

**Table 5:** Visual Acuity and Central Macular Thickness at 18-Month Follow-Up

	Group A (*N* = 19)	Group B (*N* = 19)	Total (*N* = 38)	*P*
Best-Corrected Visual Acuity- 18 m				< 0.0011
Mean ± SD	0.17 ± 0.08	0.34 ± 0.11	0.26 ± 0.12	
Range	0.1–0.3	0.1–0.5	0.1–0.5	
CMT-OCT µm − 18 m				< 0.001
Mean ± SD	239.5 ± 12.5	263.2 ± 19.8	251.4 ± 20.1	
Range	228.0–265.0	230.0–300.0	228.0–295.0	

CMT: Central Macular Thickness, OCT: Optical Coherence Tomography, SD: Standard Deviation

### Complications

Ocular adverse events were rare in both groups, as seen in Table 6. The BRZ group had a greater incidence of uveitis (26.3%) than the AFL group (15.8%), although the difference was not statistically significant (*p* = 0.426). A single case of mild retinal vasculitis (5.3%) occurred in the BRZ group, with no such events reported in the AFL group (*p* = 0.487). The affected patient responded well to systemic and topical corticosteroid therapy, and subsequent anti-VEGF treatment was switched without further complications. No cerebrovascular or systemic adverse events, including stroke, were reported in either group (*p* = 1.000), indicating a comparable systemic safety profile.

**Table 6:** Post-Treatment Ocular and Systemic Complications

	Group A (*N* = 19)	Group B (*N* = 19)	Total (*N* = 38)	*P*
Uveitis				0.4261
Yes	5.0 (26.3%)	3.0 (15.8%)	8.0 (21.1%)	
No	14.0 (73.7%)	16.0 (84.2%)	30.0 (78.9%)	
Vasculitis				0.4866
Yes	1.0 (5.3%)	0.0 (0.0%)	1.0 (2.6%)	
No	18.0 (94.7%)	19.0 (100.0%)	37.0 (97.4%)	
Stroke				1.0
Yes	0 (0%)	0 (0%)	0 (0%)	
No	19.0 (100%)	19 (100%)	38.0 (100%)	

## Correlation and regression analysis

**Fig. 1:**
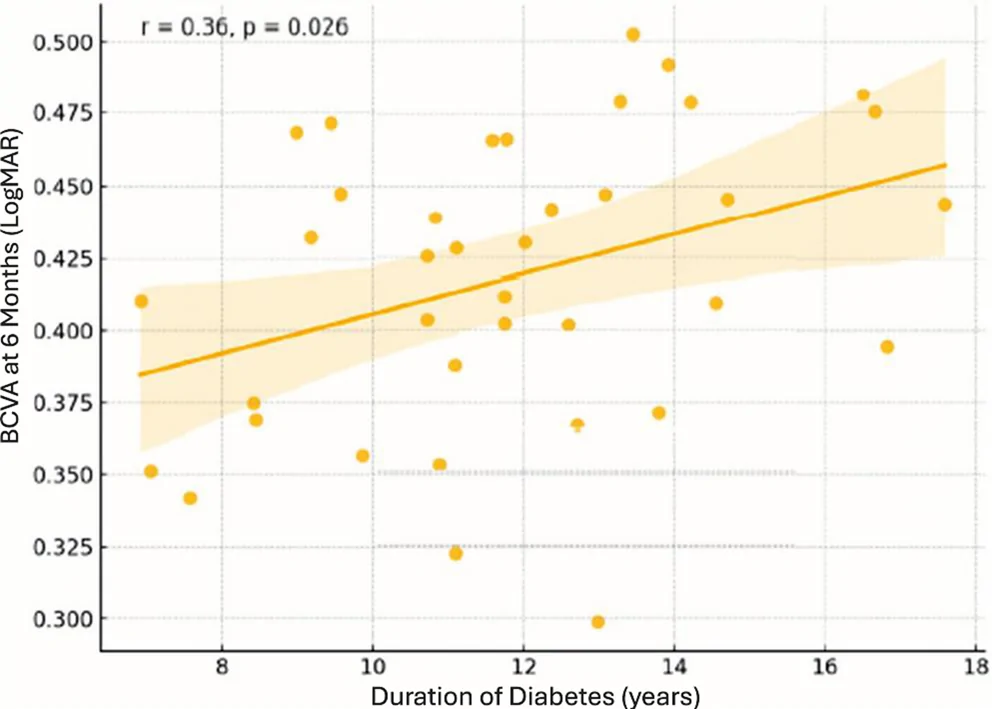
Correlation between Duration of Diabetes and BCVA at 6 Months

**Fig. 2:**
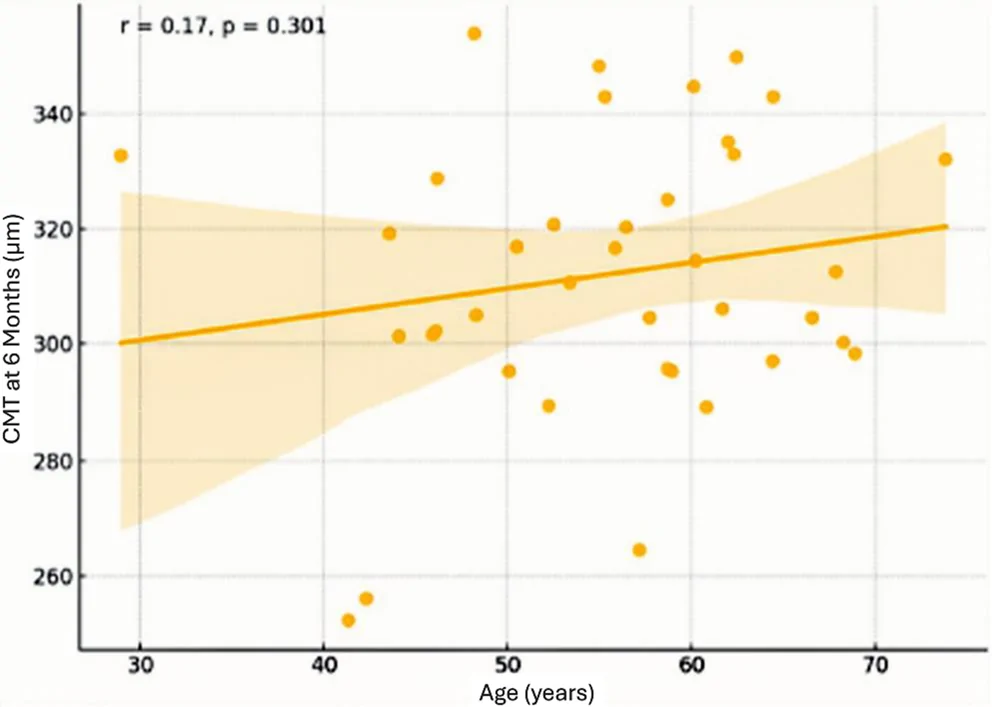
Correlation between Age and CMT-OCT at 6 Months

**Fig. 3:**
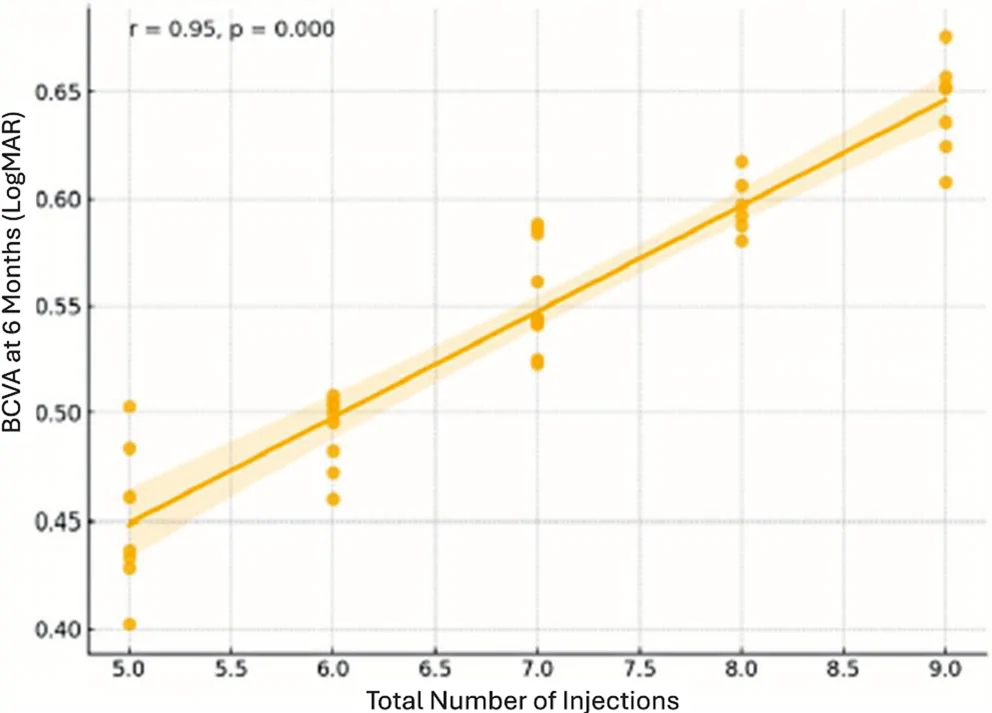
Correlation between BCVA at 6 Months and Total Number of Injections

Correlation analyses are illustrated in Figs. 1, 2 and 3. A weak but statistically significant correlation was observed between the duration of diabetes and BCVA at 6 months (*r* = 0.36, *p* = 0.026), suggesting that a longer diabetic history may modestly influence visual outcomes. In contrast, a weak, non-significant correlation was found between age and CMT at 6 months (*r* = 0.17, *p* = 0.301), indicating minimal age-related effect on anatomical response.

The total number of injections and BCVA at 6 months showed a strong, statistically significant positive connection (*r* = 0.95, *p* < 0.001), highlighting the importance of injection frequency in maximizing visual recovery.

Higher intraocular pressure (β = 0.016; 95% CI 0.003–0.029; *p* = 0.015) and a better baseline BCVA (β = 0.165; 95% CI 0.040–0.289; *p* = 0.011) were independently linked to greater visual improvement at 6 months, according to multivariable linear regression analysis (Table 7). While the total number of injections had the biggest favorable impact (β = 0.173; 95% CI 0.102–0.244; *p* < 0.001), central macular thickness had an adverse relationship with BCVA (β = − 0.00088; 95% CI − 0.00160 to − 0.00017; *p* = 0.017). Variables including age, sex, and duration of diabetes were not significant predictors of visual outcome.

**Table 7:** Linear Regression Analysis for Predictors of BCVA-OD at 6 Months

Predictor	Estimate (*r*)	95% CI Lower	95% CI Upper	*p*-value
Age	0.003	-0.001	0.007	0.139
Sex (Male vs. Female)	-0.023	-0.094	0.048	0.512
Duration of DM (years)	0.002	-0.010	0.015	0.698
Intraocular Pressure (mmHg)	0.016	0.003	0.029	0.015*
Best-Corrected Visual Acuity (Baseline)	0.165	0.040	0.289	0.011*
CMT-OCT µm	-0.00088	-0.00160	-0.00017	0.017*
Total No. of injections	0.173	0.102	0.244	< 0.001**

CMT: Central Macular Thickness, OCT: Optical Coherence Tomography, SD: Standard Deviation

## Discussion

In this retrospective cohort study of DME patients with CMT > 350 μm, brolucizumab (BRZ) required significantly fewer intravitreal injections (mean = 5) than aflibercept (AFL) (mean = 9), representing a 44.4% reduction in treatment burden over 18 months. This directly correlates with our results and supports the study objective of evaluating efficacy alongside injection burden. The finding is consistent with clinical practice–based studies demonstrating BRZ’s extended dosing potential. A prospective observational study from the Berlin Macula Registry reported that at week 36, BRZ achieved comparable improvements in BCVA and central retinal thickness (CRT) to AFL in DME eyes, with mean treatment intervals of 11.3 weeks versus 6.5 weeks, respectively (12). Similar trends were noted in previously treated eyes (9.3 vs. 5.3 weeks), emphasizing BRZ’s capacity to maintain efficacy with fewer injections [12, 13].

Our observed injection frequency (5 vs. 9) corresponds well with a 12-month case series by Elhamaky et al. (2024), which reported means of 6.3 ± 0.45 for BRZ and 7.25 ± 0.53 for AFL (*p* < 0.0001) [14]. The greater reduction in our study suggests increased dosing flexibility in clinical practice. Similarly, the BRADIR study (2023) showed that BRZ produced significant visual and anatomical improvements in recalcitrant DME with fewer injections, whereas AFL demonstrated limited gains at 52 weeks [15]. Our 18-month data confirm this pattern: BRZ achieved superior BCVA (0.17 ± 0.08 vs. 0.34 ± 0.11; *p* < 0.001) and greater CMT reduction (239.5 ± 12.5 μm vs. 263.2 ± 19.8 μm; *p* < 0.001) despite fewer injections. These findings reinforce BRZ’s sustained efficacy and durability.

BRZ’s low molecular weight and high molar dose enable prolonged VEGF suppression, supporting extended dosing intervals [3, 8]. Our results—showing resolution of macular edema with fewer injections—corroborate the BRADIR study’s observation of sustained anatomical improvement [15]. The KINGFISHER trial similarly found BRZ (6 mg) to be non-inferior to AFL (2 mg) for BCVA gains in DME [16]. Thus, our retrospective comparative results extend these clinical trial findings, highlighting the practical advantage of BRZ’s longer durability and deeper tissue penetration [7].

The clinical implications of BRZ’s reduced injection frequency are substantial. Fewer injections can enhance patient adherence, reduce procedure-related risks (e.g., endophthalmitis), and minimize treatment burden, particularly for elderly or comorbid patients [12, 15]. These advantages are especially valuable in resource-limited or high-volume clinical settings. Real-world data—including the Berlin Macula Registry and BRADIR—support the notion that extended BRZ dosing intervals improve compliance without compromising efficacy [12, 15]. Our study reinforces BRZ’s value as a patient-centered, efficient therapy for DME.

A notable safety observation was the low incidence of retinal vasculitis in the BRZ group (5.3%, 1/19) compared with none in the AFL group (*p* = 0.4866), which was not statistically significant. This rate is consistent with real-world reports (2.4–16%) and comparable to the < 1% incidence observed in clinical trials such as KINGFISHER (0.9% for BRZ vs. 0.6% for AFL) [9, 10, 15]. Our lower rate likely reflects the exclusion of patients with pre-existing vasculitis or autoimmune disorders (e.g., SLE). The single affected patient developed mild inflammation without vision loss and responded completely to prompt corticosteroid therapy (oral prednisolone 1 mg/kg/day + topical prednisolone acetate 1%). This highlights the importance of early recognition, immediate anti-inflammatory intervention, and close monitoring to ensure safety.

### Clinical significance and future directions

The superior BCVA and CMT outcomes, combined with a 44.4% reduction in injection frequency, underscore BRZ’s role in optimizing DME management. Linear regression analysis identified injection number, baseline BCVA, and CMT as significant predictors of visual improvement, emphasizing the importance of treatment adherence. These results align with clinical practice–based studies such as the Berlin Macula Registry and BRADIR, which confirmed sustained functional gains with extended BRZ dosing [12, 15]. The reduced treatment burden also decreases clinic visits, caregiver dependence, and healthcare costs [17].

To confirm these results, evaluate long-term safety (especially vasculitis risk), and analyze patient-reported outcomes like satisfaction, quality of life, and cost-effectiveness across various healthcare systems, prospective, multicenter studies with standardized treatment protocols are required.

## Strengths and limitations

This study has a number of noteworthy advantages. In centers with diabetic macular edema (DME), it offers comparative data on brolucizumab and aflibercept that is based on actual clinical decision-making and patient care practices outside of controlled study settings. The follow-up period of 18 months allowed for meaningful assessment of both short-term and sustained anatomical and visual outcomes, as well as the incidence of ocular inflammation. The inclusion of well-defined baseline and follow-up parameters—such as BCVA, CMT, and injection burden—enables comprehensive evaluation of treatment efficacy and durability. Furthermore, all imaging and assessments were performed by the same clinical team using standardized OCT and BCVA protocols, minimizing interobserver variability.

However, this study also has limitations. The retrospective, non-randomized design introduces inherent risks of selection and information bias, despite comparable baseline characteristics between groups. The difference in loading regimens—three bimonthly injections for BRZ versus five monthly injections for AFL—represents a major confounding factor that may have influenced injection frequency and early treatment response. Nevertheless, this regimen reflects current actual clinical practice, where dosing intervals are individualized according to the pharmacologic durability of each anti-VEGF agent.

Additionally, a significant baseline sex imbalance (68.4% males in Group A vs. 68.4% females in Group B, *p* = 0.0232) may have influenced inflammatory outcomes, as female sex has been associated with higher susceptibility to vasculitis. The non-standardized “pro re nata” (PRN) criteria used to guide retreatment could also have affected injection frequency comparisons. Finally, the relatively small sample size (*N* = 38), single-center design, and 18-month follow-up duration limit the generalizability of these findings. Larger, prospective, multicenter studies with standardized treatment protocols are warranted to confirm the long-term efficacy, safety, and cost-effectiveness of BRZ in diabetic macular edema.

## Conclusion

Both aflibercept (AFL) and brolucizumab (BRZ) successfully decreased central macular thickness (CMT) and enhanced best-corrected visual acuity (BCVA) in eyes with center-involving diabetic macular edema (DME), according to this retrospective comparative analysis. BRZ demonstrated significantly greater visual and anatomical improvement compared with AFL at 12 and 18 months, while requiring significantly fewer injections during the 18-month follow-up, indicating a reduced treatment burden. A slightly higher, yet manageable, incidence of intraocular inflammation was noted in the BRZ group, consistent with findings from prior real-world studies.

Overall, BRZ appears to offer comparable or superior anatomical efficacy to AFL with fewer injections, supporting its consideration as a viable therapeutic option for DME in clinical practice. However, due to the retrospective design, small sample size, and non-standardized retreatment criteria, care should be used when interpreting these findings. To confirm the long-term effectiveness and safety of BRZ in DME therapy, prospective, randomized multicenter trials with defined treatment regimens are necessary.

## Data Availability

All data supporting the results and conclusions of this study have been included in the revised manuscript. No additional datasets were generated or analyzed in this study. However, further details may be made available from the corresponding author upon reasonable request, subject to institutional and ethical approval, where applicable.

## Abbreviations

AFL: Aflibercept
Anti-VEGF: Anti–vascular endothelial growth factor
BCVA: Best-corrected visual acuity
BRZ: Brolucizumab
CMT: Central macular thickness
DME: Diabetic macular edema
ETDRS: Early Treatment Diabetic Retinopathy Study
IOP: Intraocular pressure
logMAR: Logarithm of the minimum angle of resolution
OCT: Optical coherence tomography
PRN: Pro re nata (as needed)
SD: Standard deviation

## References

[1] Ko S (2023) Productivity loss among patients with diabetic macular edema in two eyes: a retrospective commercial claims analysis in the United States. University of Washington

[2] Hachana S, Larrivée B (2022) TGF-β Superfamily Signaling in the Eye: Implications for Ocular Pathologies. Cells Jul 29(15). 10.3390/cells11152336 PMC9367584 35954181

[3] Sydnor S, Chatterjee S, Cooney P et al (2023) Efficacy and Safety of Brolucizumab, Aflibercept, and Ranibizumab for the Treatment of Patients with Visual Impairment Due to Diabetic Macular Oedema: A Systematic Review and Network Meta-Analysis. Diabetes Ther Jul 14(7):1193–1216. 10.1007/s13300-023-01410-8 PMC10241757 37198521

[4] Brown DM, Emanuelli A, Bandello F et al (2022) KESTREL and KITE: 52-Week Results From Two Phase III Pivotal Trials of Brolucizumab for Diabetic Macular Edema. Am J Ophthalmol Jun 238:157–172. 10.1016/j.ajo.2022.01.004 35038415

[5] Wykoff CC, Garweg JG, Regillo C et al (2024) KESTREL and KITE Phase 3 Studies: 100-Week Results With Brolucizumab in Patients With Diabetic Macular Edema. Am J Ophthalmol Apr 260:70–83. 10.1016/j.ajo.2023.07.012 37460036

[6] Arora A, Morya AK, Gupta PC, Menia NK, Nishant P, Gupta V (2024) Intravitreal therapy for the management of diabetic retinopathy: A concise review. World J Exp Med Dec 20(4):99235. 10.5493/wjem.v14.i4.99235 PMC11551706 39713073

[7] Chakraborty D, Mondal S, Parachuri N, Kumar N, Sharma A (2022) Brolucizumab-early experience with early extended interval regime in chronic centre involved diabetic macular oedema. Eye (Lond) Feb 36(2):358–360. 10.1038/s41433-021-01816-3 PMC8807620 34645970

[8] Abu Serhan H, Taha MJJ, Abuawwad MT et al (2024) Safety and Efficacy of Brolucizumab in the Treatment of Diabetic Macular Edema and Diabetic Retinopathy: A Systematic Review and Meta-Analysis. Semin Ophthalmol May 39(4):251–260. 10.1080/08820538.2023.2271095 37849309

[9] Khanani AM, Zarbin MA, Barakat MR et al (2022) Safety Outcomes of Brolucizumab in Neovascular Age-Related Macular Degeneration: Results From the IRIS Registry and Komodo Healthcare Map. JAMA Ophthalmol Jan 1(1):20–28. 10.1001/jamaophthalmol.2021.4585 PMC8613703 34817566

[10] Radke NV, Mohamed S, Brown RB et al (2023) Review on the Safety and Efficacy of Brolucizumab for Neovascular Age-Related Macular Degeneration From Major Studies and Real-World Data. Asia Pac J Ophthalmol (Phila) 12(2):168–183. 10.1097/apo.0000000000000602 36971706

[11] Bragança F, Ferreira A, Leite J et al (2024) Real-World Experience With Brolucizumab 6 mg for Diabetic Macular Edema. Cureus Jan 16(1):e52176. 10.7759/cureus.52176 PMC10859174 38344619

[12] Rübsam A, Hössl L, Rau S, Böker A, Zeitz O, Joussen AM (2024) Real-World Experience with Brolucizumab Compared to Aflibercept in Treatment-Naïve and Therapy-Refractory Patients with Diabetic Macular Edema. J Clin Med Mar 21(6). 10.3390/jcm13061819 PMC10970839 38542043

[13] Charters L Brolucizumab shows promise over aflibercept for diabetic macular edema in meta-analysis

[14] Elhamaky TR (2024) Comparison between intravitreal brolucizumab and aflibercept in the treatment-naive central involved diabetic macular edema: One-year real-life case series. Eur J Ophthalmol May 34(3):797–802. 10.1177/11206721231207459 37817540

[15] Chakraborty D, Sharma A, Mondal S et al (2024) Brolucizumab versus aflibercept for recalcitrant diabetic macular edema in Indian real-world scenario - The BRADIR study. Am J Ophthalmol Case Rep Dec 36:102152. 10.1016/j.ajoc.2024.102152 PMC11387804 39263686

[16] Singh RP, Barakat MR, Ip MS et al (2023) Efficacy and Safety of Brolucizumab for Diabetic Macular Edema: The KINGFISHER Randomized Clinical Trial. JAMA Ophthalmol Dec 1(12):1152–1160. 10.1001/jamaophthalmol.2023.5248 PMC12512174 37971723

[17] Yu JS, Carlton R, Agashivala N, Hassan T, Wykoff CC (2021) Brolucizumab vs aflibercept and ranibizumab for neovascular age-related macular degeneration: a cost-effectiveness analysis. J Manag Care Spec Pharm Jun 27(6):743–752. 10.18553/jmcp.2021.27.6.743 PMC10390922 34057392

